# Obituary Notices

**Published:** 1864-06

**Authors:** 


Death of Dr. Cheney.—At the meeting of the Chicago Medical Society, on Friday evening, May 6th, the following resolutions were adopted:

*Resolved*, That in the death of L. P. Cheney, our Medical Society has lost a highly esteemed member, our profession a laborious and conscientious practitioner, and our community a warm-hearted, useful and noble man.

*Resolved*, That while we deeply mourn our loss, we tender our heart-felt sympathies to the bereaved family and relatives and large circle of friends.

*Resolved*, That a copy of the foregoing be transmitted to the family of the deceased, also to the daily city papers, the *Chicago Medical Journal*, and the *Medical Examiner*, for publication, and that they be transcribed upon the records of this Society.

J. P. Ross, M. D.,

H. W. Jones, M. D., Orren Smith, M. D., } Committee.

———

John Redman Coxe, M. D.—Dr. Coxe was the oldest graduate of the Medical Department of the University of Pennsylvania, in which institution he was for many years a Professor.

He was born in Trenton, New Jersey, on the 16th of September, 1773. He was educated in Philadelphia, under the charge of his grandfather, Dr. John Redman, until his tenth year, when he went to England, where he remained at school until his seventeenth year, when he went to Edinburgh to complete his classical education; while there he attended a course of medical lectures at the University. He returned to America in 1790, and at once commenced the regular study of medicine with Dr. Benjamin Rush, with whom he remained until 1794, when he received his diploma. While with Dr. Rush he was actively engaged in practice during the severe visitation of the yellow fever in 1793, at which time three of his five fellow-students died of the fever. Immediately after graduating he went to London, and became a house-pupil at the London Hospital, and remained there nearly a year. He then went to Edinburgh, and attended a course of lectures at the University; thence to Paris, where he pursued his medical studies for three months. He then returned to London, where he spent several months in the hospitals. He returned to the United States, and settled in Philadelphia in the winter of 1796–7, when he at once entered upon the active practice of the profession for which he had been so carefully preparing himself by many years of study.

Dr. Coxe was appointed, by the Board of Health, physician to the port during the second visitation of the yellow fever, in 1798. He was for several years one of the physicians of the Pennsylvania Hospital, and also of the Philadelphia Dispensary. He was largely engaged in private practice, when, in 1809, he was elected Professor of Chemistry in the University of Pennsylvania, from which chair he was transferred, in 1818, to that of Materia Medica and Pharmacy, which he held until 1835. For many years he has been leading the quiet and retired life of a student. Dr. Coxe was one of the earliest introducers of vaccination into the United States, and was the first to introduce it into this city. His name has for more than half a century been a household word in connection with the Hive Syrup (*Syrupus Scillæ Compositus*, U. S. P.) which he invented, and which has proved such an inestimable blessing to thousands. He has passed away quietly, without disease, at the advanced age of ninety years and six months, having never been sick in all that time.—*Reporter.*

DEATH OF DR. ROBERT P. THOMAS. We observe with sorrow, the death of this gentleman noted in the Philadelphia *Med. and Surg. Reporter*. He was the editor of the last edition of *Ellis' Formulary*, a notice of which is in the present number of the *Press*. A singular mortality has attended this work, Ellis, its author, and Morton and Thomas, successively its edtors, having passed away in the prime of life.—*San Francisco Med. Press*.

---

DEATH OF PROFESSOR RIBES.—M. Ribes, professor of hygiene at the Faculty of Montpellier, has lately died at Perpignan, his native place, at the age of sixty-three. The deceased, published, three years ago, an important work entitled "Therapeutical Hygiene," and had previously enriched medical literature with valuable works, essays, and speeches.—*Lancet*.

---

*City Mortality*.—The following is the mortality of Chicago for six years past:

| | 1858. | '59. | '60. | '61. | '62. | '63. |
|---|---|---|---|---|---|---|
| January, | 117 | 131 | 115 | 193 | 174 | 321 |
| February, | 126 | 115 | 128 | 135 | 197 | 294 |
| March, | 156 | 132 | 180 | 172 | 199 | 298 |
| April, | 116 | 155 | 131 | 126 | 187 | 300 |
| May, | 139 | 115 | 102 | 134 | 166 | 257 |
| June, | 139 | 112 | 155 | 131 | 156 | 219 |
| July, | 268 | 164 | 133 | 239 | 274 | 414 |
| August, | 377 | 325 | 308 | 262 | 304 | 341 |
| September, | 238 | 190 | 172 | 227 | 271 | 323 |
| October, | 102 | 160 | 149 | 120 | 246 | 258 |
| November, | 136 | 102 | 170 | 155 | 196 | 220 |
| December, | 135 | 125 | 158 | 195 | 203 | 230 |
| Total, | 2,049 | 1,826 | 2,056 | 2,069 | 2,575 | 3,475 |

---

*Chronic Ulcers*.—Dr. Skey says, "I have treated a large number of these affections, and with success. The more chronic the ulcer, the larger its size, the more aged the subject, the more remarkable is the influence of opium in effecting a cure. Let a case be selected for experiment, of some twenty years' duration, which has exhausted the patience of various medical attendants, as well as the remedies employed by them for cure.

"Treat such a case of chronic ulcer, of the largest size, having a pale, flat, bloody base, a high mound lymped around it, covered by healthy integument, the sore pouring out large quantities of watery ichor, saturating every covering. Select such a case occurring in old age: give such a person ten to fifteen erops of tincture of opium night and morning, leaving the bowels alone, and observe the base of the sore in five or six days: it will exhibit a number of minute red points, which, daily increasing in number, will rise up in the form and identity of healthy granulations, and cover the entire surface of the ulcer; and at the same time the base is becoming elevated, the margin becomes depressed, and the process of cicatrization is commenced.

"No injury to the constitution attaches to the use of this remedy, its salutary action upon the ulcer is obtained solely through the healthy influence it exercises upon the constitution."—*London Lancet.*

---

*Death From Chloroform, in San Francisco.*—Mrs. ——, a native of California, of highly nervous temperament, went to a dentist's to have a tooth extracted. She had a great dread of the instruments, and while much excited, chloroform was administered, by holding a napkin above and near the nostrils. She had some difficulty in managing the respiration, and, after inhaling it imperfectly a short time, complained that she could not take it, and seized the hand of the operator, removing it from her face. Without using the chloroform any longer, her hands were held, and the tooth extracted without difficulty. Immediately after the withdrawal of the forceps from her mouth, her jaws were clenched and her head thrown back, by spasmodic contraction of the muscles. The breathing was arrested, the face grew livid, and death rapidly ensued. The immediate cause of death appears to have been apnœa, from the spasmodic closure of the glottis or larynx. The masseter, respiratory and spinal muscles, and also the flexors of the extremities, continued in a state of tonic spasm till relaxed by death. We are satisfied that there was no mismanagement of chloroform in this case—unless to use it at all be a mismanagement. There is quite as much reason to allege that it was not pushed far enough, as that it was pushed too far. Possibly a longer inhalation would have carried her safely beyond the range of spasm, and into the tranquil region of anæsthesia. There was no disease of the heart or other organs to discourage the use of the agent.—*San Francisco Med. Press.*

